# Giant Biatrial Myxoma Presenting as Constitutional Symptoms and Bivalvular Obstruction

**DOI:** 10.1016/j.jaccas.2026.107406

**Published:** 2026-03-16

**Authors:** Ammar Akhtar, Abdullah Hashmi, Ifrah Tahir, Kashif Ali Hashmi

**Affiliations:** Chaudhary Pervaiz Elahi Institute of Cardiology, Multan, Pakistan

**Keywords:** hemodynamics, mitral valve, tricuspid valve

## Abstract

**Background:**

Simultaneous biatrial myxoma is an extremely rare form of primary cardiac tumor, which is often an acute life-threatening problem given its potential to cause simultaneous tricuspid and mitral valve obstructions.

**Case Summary:**

A 25-year-old woman presented with a history of persistent low-grade fever, 6 kg of weight loss, and progressive dyspnea for 6 months. Transthoracic echocardiography showed a large mass from the interatrial septum and right atrial wall originating from the left atrium. This giant tumor was causing severe tricuspid obstruction and severe narrowing of the mitral valve. There was evidence of associated pulmonary emboli on computed tomography and computed tomography pulmonary angiography as well. The patient underwent successful urgent surgical excision.

**Discussion:**

This case describes the rare presentation of giant biatrial myxoma in the form of chronic systemic illness. It emphasizes the role of early cardiac imaging in young patients with unexplained constitutional symptoms and features of biventricular heart failure.

**Take-Home Messages:**

Giant biatrial myxoma is very rare and is life threatening. Prompt and definitive surgical resection results in good outcome and is the treatment of choice.

## Graphical Abstract

**Figure:**
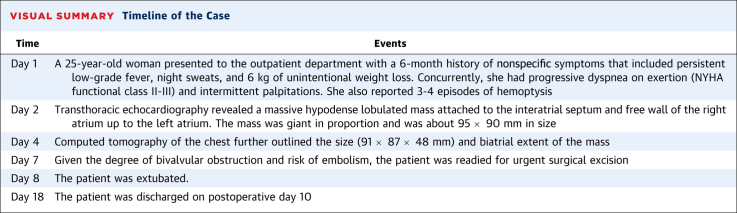

## History of Presentation

A 25-year-old woman presented to the outpatient department with a 6-month history of nonspecific symptoms that included persistent low-grade fever, night sweats, and 6 kg of unintentional weight loss. Concurrently, she had progressive dyspnea on exertion (NYHA functional class II-III) mentioning symptomatic relief with change of posture, as well as intermittent palpitations. She also reported 3 to 4 episodes of hemoptysis. On physical examination she was tachycardic (122 beats/min) and had signs of chronic right-sided heart failure including an elevated jugular venous pressure and mild tenderness over the right upper quadrant. Auscultation revealed the presence of a prominent diastolic “tumor plop” sound and holosystolic murmur over the tricuspid area.

**Take-Home Messages**

• Giant biatrial myxoma is very rare and is life threatening.
• Prompt and definitive surgical resection results in good outcome and is the treatment of choice.

## Past Medical History

The patient had undergone 2 courses of antibiotics in the past and had a negative work-up for pulmonary tuberculosis, which is a common differential diagnosis in the region. Other than that, she had no significant past medical history.

## Differential Diagnosis

The initial presentation had provided a number of potential underlying diagnoses. The most likely diagnosis was cardiac tumor (myxoma) given diastolic tumor plop (classic for a mobile mass prolapsing through the valve), positional dyspnea, weight loss, and signs of right-sided heart failure. Other possible causes were large mobile atrial thrombus or infective endocarditis.

## Investigations

Initial laboratory results were remarkable for mild elevation in liver enzymes: aspartate aminotransferase 133 U/L (normal: <40 U/L) and total bilirubin 1.8 mg/dL (normal: 1.2 mg/dL), consistent with hepatic congestion. Hemoglobin, white blood cell counts, and C-reactive protein were within normal limits.

Transthoracic echocardiography revealed a massive hypodense lobulated mass attached to the interatrial septum and the free wall of the right atrium up to the left atrium. The mass was giant in proportion, and was about 95 × 90 mm in size (Videos 1 and 2). The tumor caused significant mechanical obstruction owing to distension of the right atrium and prolapse into the right ventricle during diastole and severe tricuspid valve obstruction. The mass squashed the left atrium and caused severe narrowing of the mitral valve orifice. This dual obstruction was linked to important retrograde findings such as severe dilation of the inferior vena cava and hepatic veins.

**Figure 1:**
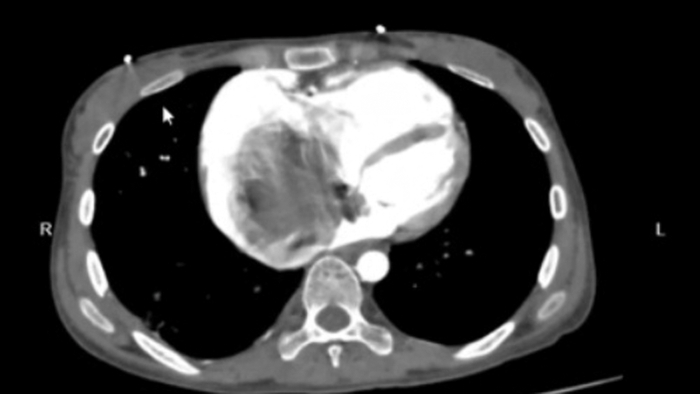
Large Filling Defect/Mass Adherent to the Interatrial Septum, Right Atrial Wall, and Endocardial Cushion Extending Into the Left Atrium

Computed tomography (CT) of the chest was performed, which further outlined the size (91 × 87 × 48 mm) and biatrial extent of the mass (Figure 1). The CT findings were also suggestive of multiple subsegmental bilateral pulmonary emboli with peripheral consolidative opacities, a rare but life-threatening embolic complication.

## Intervention and Outcome

**Figure 2:**
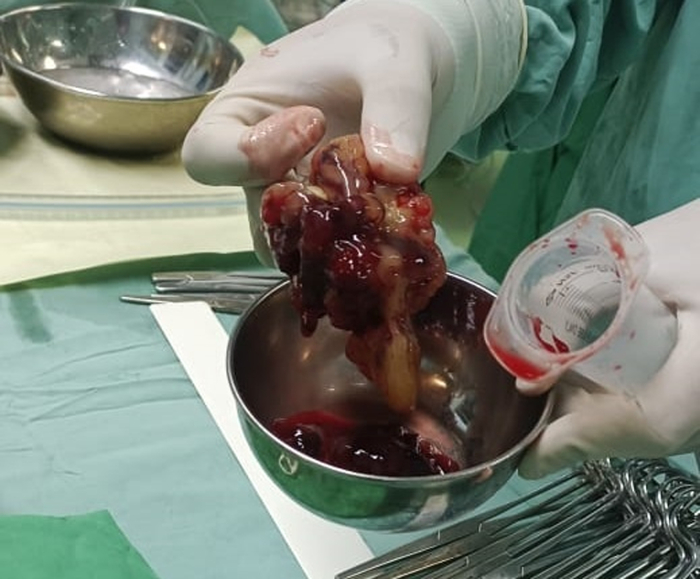
Whole of the Mass Resected Successfully: Gelatinous and Polylobulated Aspect of the Myxoma

Given the degree of bivalvular obstruction and risk of embolism, the patient was readied for urgent surgical excision. Through a median sternotomy, and by cardiopulmonary bypass, the heart was arrested. The right atriotomy revealed the large and fragile gelatinous mass extending from the right atrium into the interatrial septum. A transseptal approach was used in order to ensure removal of the portions continuing into the left atrium. The entire mass was successfully removed (Figure 2). After resection, the hemodynamic status of the patient was quickly stabilized. The levels of liver enzymes were back to normal after 1 week. Histopathological examination confirmed the gross findings of a benign cardiac myxoma made up of stellate cells in a myxoid stroma with a high content of acid mucopolysaccharides. The patient was discharged on postoperative day 10 with improvement of her constitutional symptoms and dyspnea.

## Discussion

This case is a rare manifestation of a cardiac myxoma for a number of reasons. First, biatrial myxomas are uncommon in incidence (<5%).^1^ Second, the size of the tumor was massive (maximum dimension: 9.1 cm), and therefore qualified as “giant myxoma.” The literature on giant atrial myxoma is dominated by case reports emphasizing its rare occurrence rather than its true incidence.^2^ Third, the presentation with 6 months of ill-defined fever, weight loss, and elevated hepatic enzymes, as if they were chronic inflammatory or infectious diseases, is a classic but frequently misleading systemic presentation of myxomas because of the production of inflammatory cytokines.^3^ The clinical course of the patient was dominated by the presence of severe obstruction of bivalvular inflow (tricuspid and mitral) as hemodynamic sequelae unique to giant biatrial masses. This impingement from both directions led to rapid progression of dyspnea and congestion including hepatic dysfunction. The positional component of her dyspnea is also suggestive of the mobility of the tumor plug. Furthermore, the CT evidence of pulmonary emboli bears out the inherent risk of tumor fragmentation and embolization, which is particularly high with large and friable myxomas.^4^ Early diagnosis on transthoracic echocardiography was critical. This modality not only detected the mass but gave precise information about its size and its location of attachment to the interatrial septum as well as the life-threatening obstruction of both of the atrioventricular valves.

Given the high-risk for sudden cardiac death caused by hemodynamic collapse and massive embolization, urgent surgical excision is mandatory at diagnosis based on tumor size and symptom severity.^5^ The biatrial approach associated with wide excision used in the present study is the standard approach to ensure complete tumor removal and reduce the risk of local recurrence,^6^^,^^7^ especially when a septal, multichambered mass is present. This case raises the importance of a high index of suspicion for cardiac tumors in patients with constitutional symptoms and evidence of unexplained heart failure, even after the exclusion of all forms of endemic infectious disease.

## Conclusions

Giant biatrial myxoma is a very rare condition that needs to be treated immediately. The symptomatology presentation with protracted constitutional symptoms and critical bivalvular obstruction emphasizes the functional malignancy of these biologically benign tumors. Prompt diagnosis with echocardiography and prompt full surgical resection with biatrial approach is an excellent cure and prognosis.

## Funding Support and Author Disclosures

The authors have reported that they have no relationships relevant to the contents of this paper to disclose.

## Appendix

Video 1 Transthoracic Echocardiography: Demonstrating Biatrial Myxoma Apical 4-chamber view showed 2 distinct, mobile intracardiac masses arising from the interatrial septum, protruding into both the right and left atria, consistent with a rare biatrial myxoma. The tumors demonstrated heterogeneous echogenicity and significant mobility during the cardiac cycle.

Video 2 Transthoracic Echocardiography (Apical 4-Chamber View) Showing Atrial Myxoma on Right Side Apical 4-chamber view demonstrated a large, well-defined right atrial mass with increased clarity on the right-sided chambers, protruding into the right ventricle during diastole and causing functional obstruction at the tricuspid valve.
